# Supplementary material for: Tracing frequent users of regional care services using emergency medical services data: a networked approach
Source: BMJ Open. 2020 May 27;10(5):e036139. doi: 10.1136/bmjopen-2019-036139 (PMC7259845; doi:10.1136/bmjopen-2019-036139)
Supplement: Author's manuscript [file bmjopen-2019-036139.draft_revisions.pdf]

BMJ Open is committed to open peer review. As part of this commitment we make the peer review history of every article we publish publicly available.

When an article is published we post the peer reviewers' comments and the authors' responses online. We also post the versions of the paper that were used during peer review. These are the versions that the peer review comments apply to.

The versions of the paper that follow are the versions that were submitted during the peer review process. They are not the versions of record or the final published versions. They should not be cited or distributed as the published version of this manuscript.

BMJ Open is an open access journal and the full, final, typeset and author-corrected version of record of the manuscript is available on our site with no access controls, subscription charges or pay-per-view fees (http://bmjopen.bmj.com).

If you have any questions on BMJ Open's open peer review process please email info.bmjopen@bmj.com

BMJ Open

# Tracing frequent users of regional care services - a networked approach


| | |
|---|---|
| Journal: | *BMJ Open* |
| Manuscript ID | bmjopen-2019-036139 |
| Article Type: | Original research |
| Date Submitted by the Author: | 02-Dec-2019 |
| Complete List of Authors: | Maruster, Laura; University of Groningen Faculty of Economics and Business, IM&S<br>van der Zee, Durk-Jouke; Rijksuniversiteit Groningen Faculteit Economie en Bedrijfskunde, Operations<br>Hatenboer, Jaap; UMCG, Ambulancezorg<br>Buskens, Erik; University of Groningen, University Medical Center Groningen, Epidemiology |
| Keywords: | Health policy < HEALTH SERVICES ADMINISTRATION & MANAGEMENT, ACCIDENT & EMERGENCY MEDICINE, HEALTH ECONOMICS |
| | |


SCHOLARONE™
Manuscripts

# Tracing frequent users of regional care services - a networked approach

Laura Maruster[1] PhD, Durk-Jouke van der Zee[2] PhD, Jaap Hatenboer[3], Erik Buskens[2,4] MD, PhD.

*[1] Department of Innovation Management & Strategy, Faculty of Economics & Business, University of Groningen, The Netherlands*

*[2] Department of Operations, Faculty of Economics & Business, University of Groningen, The Netherlands*

*[3] UMCG Ambulancezorg, Tynaarlo, The Netherlands*

*[4] Health Technology Assessment, Department of Epidemiology, University Medical Center Groningen, University of Groningen, Groningen, The Netherlands*

Key words: regional care services, hospital care, nursing care, emergency medical services, frequent users

* Corresponding author: Laura Maruster, Nettelbosje 2, P.O. Box 800, 9700 AV Groningen, The Netherlands. Phone + 31 50 363 7316, email: l.maruster@rug.nl

Word count: 2555

For peer review only

## ABSTRACT

**Objectives**

The aim of this study is to show how a networked approach relying on 'real-world' Emergency Medical Services (EMS) records might contribute to tracing frequent users of care services on a regional scale. Their tracing is considered of importance for policy makers and clinicians, since they represent a considerable workload and use of scarce resources. While existing approaches for data collection on frequent users tend to limit scope to individual or associated care providers, the proposed approach exploits the role of EMS as the network's "ferryman" overseeing and recording patient calls made to an entire network of care providers.

**Design**

A retrospective study was performed analysing 2012-2017 EMS calls in the province of Drenthe, The Netherlands. Using EMS data benefits of the networked approach vs. existing approaches are assessed by quantifying the number of frequent users and their associated calls for various categories of care providers. Main categories considered are hospitals, nursing homes and EMS.

**Setting**

EMS in the province of Drenthe, the Netherlands, serving a population of 491,867.

**Participants**

Analyses are based on secondary patient data from EMS records, entailing 212,967 transports and 126,758 patients, over 6 years (2012-2017).

**Results**

Use of the networked approach for analysing calls made to hospitals in Drenthe resulted in a 20% average increase of frequent users traced. Extending the analysis by including hospitals outside Drenthe increased ascertainment by 28%. Extending to all categories of care providers, inside Drenthe, and subsequently, irrespective of their location, resulted in an average increase of 132% and 152% of frequent users identified, respectively.




For peer review only - http://bmjopen.bmj.com/site/about/guidelines.xhtml

**Conclusions**

Many frequent users of care services are network users relying on multiple regional care providers, possibly representing inefficient use of scarce resources. Network users are effectively and efficiently traced by using EMS records offering high coverage of calls made to regional care providers.

For peer review only



For peer review only - http://bmjopen.bmj.com/site/about/guidelines.xhtml

## ARTICLE SUMMARY

### Strengths and limitations of this study

- The results of this study demonstrate that a networked approach for tracing frequent users relying on EMS records is capable of effectively and efficiently identifying frequent users.
- The proposed approach exploits the role of EMS as the regional 'ferryman', implying the use of a single source for data collection, covering many care providers, i.e., hospitals, nursing homes and EMS See & Treat.
- Whereas EMS records for only one province in the Netherlands are considered, it is expected that the success of the proposed approach is not dependent on this.
- As EMS records only include frequent users who are not capable of self-transport, those frequent users who do not, or rarely make use of EMS will not be traced by the proposed approach.

For peer review only



For peer review only - http://bmjopen.bmj.com/site/about/guidelines.xhtml

# INTRODUCTION

Frequent users, i.e., patients that make repetitive calls for health care services, may be responsible for a relatively large share of regional care consumption. They represent a minority of Emergency Department (ED) patients (4.5-8%), yet, they may account for up to 21-28% of all ED visits [1]. Due to their high impact on care providers' workload and associated costs they are a focal group for regional policy makers and clinicians aiming to make best use of scarce resources.

Notably, many frequent users appear to be network users, relying on multiple care providers. Their choice of care providers is influenced by, for example, their preferences and care providers' specialization. In particular, tracing patients' network use tends to be cumbersome. Hurdles not easily taken in data collection are, for example, rules on patient privacy, competition among care providers, incompatibility of information systems, and efforts to be put in. Moreover, these hurdles likely imply high data collection costs. Not surprisingly, many research designs limit their scope to single or associated care providers, with a main focus on hospitals (see for example [2] ). Hence, many factual frequent users may remain unnoticed.

Basically, current approaches towards data collection on frequent users stress probing of individual care providers [3 4]. Alternatively, acknowledging frequent users being network users, this article suggests a networked approach for their tracing, relying on EMS data. Acting as the "ferryman" in the regional network, EMS oversee and record patient calls made to regional care providers, including hospitals and nursing homes. Importantly, the EMS patient population is likely to include many frequent users [5 6]. In addition, their need for mobile nursing services and transport indicates that their requirements of care resources may be high.

The aim of the present study is to show how the use of the proposed networked approach might efficiently contribute to tracing frequent users on a regional scale.



For peer review only - http://bmjopen.bmj.com/site/about/guidelines.xhtml

# METHODS

## Care network in the province of Drenthe

The province of Drenthe has a population of 491,867 inhabitants, with a population density of 183 inhabitants per square kilometer[7]. Hospital care for its population is provided by four hospitals within the province, and by several hospitals located in neighbouring provinces. Three of the hospitals in Drenthe offer basic treatment. In one hospital the necessary skills and resources for treating multi-level traumas are present. Referral to around 80 hospitals in other provinces is motivated by reasons such as their proximity to the patient scene, patient preferences, level of care or specialization in specific treatments. Nursing care is provided by a few dozen of large homes, and around three hundred smaller (specialized) homes, mainly located within the province. EMS is provided by a single operator, relying on a network of 14 bases in 13 cities/villages in Drenthe. Its services include both urgent and planned patient transports to hospitals and planned transports to nursing homes. Planned rides are legitimated by patient care needs that prohibit self-transport.

## Data

Patient data are collected from EMS records of ambulance rides performed between January 1st 2012 – December 31st 2017. Collected data include the rides' dates and times, and destinations, i.e., care providers. EMS is marked as a formal care provider in case treatment provided by the ambulance nurse on scene suffices to address patient care needs, i.e., EMS See & Treat (EMS S&T), implying no involvement of other care providers. Motivated by EMS scope of services, three categories of care providers are distinguished, i.e., hospitals, nursing homes and EMS S&T.

**Privacy and approval**. Since the data are routinely collected for administrative purposes, and completely anonymized, i.e., there is no direct contact with identifiable persons, this study does not fall within the scope of the Medical Research Involving Human Subjects Act (WMO) [8].



For peer review only - http://bmjopen.bmj.com/site/about/guidelines.xhtml

**Patient and public involvement.** No patient and public involved.

**Data analysis**

For data analysis, EMS records referring to single rides are anonymized, cleaned by removing empty records, i.e., records not relating to patients, and inspected for correctness of data provided. Patients qualify as a frequent user if they meet a threshold of four calls in a calendar year. Although definitions differ, usually a threshold of four to five calls or more per year is used to classify a patient as a 'frequent user' [9 10]. Frequent users are quantified by presenting their numbers and number of calls, including yearly trend figures.

The potential of the proposed network-based approach for data collection on frequent users is evaluated by assessing its benefits compared to existing approaches. Whereas the proposed approach relies on EMS data, existing approaches build on data obtained from individual care providers. In principle, both approaches may render similar outcomes. However, existing approaches face hurdles not easily overcome, due to the fact that multiple organizations, i.e., care providers, are involved in data collection. Known hurdles are rules on patient privacy, competition among care providers, incompatibility of information systems, efforts to be put in, and costs of overcoming hurdles. They likely restrict the scope of data collection, i.e., the number of care providers being considered. Restriction of scope may affect identifying patients making calls to various care providers as frequent users after combining and quantifying their calls. The proposed approach relies on a single source of data, and does overcome these scoping decisions.

Effects of the choice of scope on the number of frequent users identified and their associated calls are studied by considering alternative subsets of EMS records. Choice of subsets is related to provider categories, i.e., hospitals, nursing homes, and/or EMS S&T, and their location, i.e., inside or outside Drenthe. By either allowing patient records to be combined for chosen subsets of care



For peer review only - http://bmjopen.bmj.com/site/about/guidelines.xhtml

providers, or not, beneficial effects of the possibility to identify network users are assessed. This effect is studied for hospitals, serving most of the patient calls.

## RESULTS

EMS records for 2012-2017, refer to 212,967 calls for services, involving 126,758 patients. Data cleaning resulted in 2,494 calls being removed. In addition, 13,156 calls were discarded due to their lack of information on transport destination, i.e., care provider.  The remaining 199,811 calls are included in the study. Out of these 199,811 calls 147,027 (74%), 10,976 (5%), and 41,808 (21%) refer to services provided by hospitals, nursing homes, and EMS S&T, respectively.

Results of the evaluation of the networked approach for data collection on frequent users are shown in Tables 1-4. Tables 1 and 2 quantify the number of *frequent users* and their associated *calls* for alternative choices of categories of care providers located in Drenthe on a yearly basis. Categories of care providers considered are hospitals, nursing homes, EMS S&T, and all care providers, i.e., taking all aforementioned categories together. Except for hospitals, all results assume data sharing among care providers within categories set, allowing frequent users being network users to be traced. In addition, hospitals results are shown for settings where such data sharing among single hospitals is not possible. Hence, network users may be neglected. For respective settings, the number of unique frequent users is shown, i.e., numbers are corrected for the fact that a single patient may be classified as a frequent user for multiple hospitals. The final column indicates the effect of combining data for all care providers vs. a setting where frequent users of hospital services are identified by studying single hospitals in isolation. It shows how the number of frequent users traced and their associated calls increase by more than a twofold by combining data for all care providers. Similar to Tables 1 and 2, Tables 3 and 4 quantify the annual number of *frequent users* and their associated *calls* for alternative choices of categories of care providers, without setting



For peer review only - http://bmjopen.bmj.com/site/about/guidelines.xhtml

requirements to their location. Final columns in Tables 3 and 4 show which numbers of frequent users and their associated calls are found when using the full EMS data set, including care providers located outside Drenthe. These amount to around 2.5 times the numbers found when studying hospitals in isolation.

| Year | Hospitals - no data sharing | Hospitals | Nursing homes | EMS S&T | All care providers | All care providers / Hospitals – no data shared (%) |
|---|---|---|---|---|---|---|
| 2012 | 189 | 222 | 34 | 15 | 398 | 211 |
| 2013 | 153 | 181 | 42 | 16 | 340 | 222 |
| 2014 | 204 | 245 | 22 | 19 | 495 | 243 |
| 2015 | 253 | 309 | 18 | 68 | 635 | 251 |
| 2016 | 279 | 321 | 28 | 46 | 611 | 219 |
| 2017 | 263 | 332 | 30 | 33 | 649 | 247 |

*Table 1. Number of frequent **users**, using data on care provider categories, i.e., hospitals, nursing homes, EMS S&T, and all care providers, located in Drenthe.*

| Year | Hospitals - no data sharing | Hospitals | Nursing homes | EMS S&T | All care providers | All care providers / Hospitals – no data shared (%) |
|---|---|---|---|---|---|---|
| 2012 | 1161 | 1296 | 283 | 84 | 2423 | 209 |
| 2013 | 1158 | 1279 | 497 | 73 | 2503 | 216 |
| 2014 | 1386 | 1557 | 431 | 106 | 3204 | 231 |
| 2015 | 1477 | 1711 | 174 | 388 | 3597 | 244 |
| 2016 | 1772 | 1955 | 229 | 245 | 3631 | 205 |
| 2017 | 1536 | 1821 | 193 | 170 | 3581 | 233 |

*Table 2. Number of **calls** corresponding to frequent users, using data on care provider categories, i.e., hospitals, nursing homes, EMS S&T, and all care providers, located in Drenthe.*

| Year | Hospitals – no data sharing | Hospitals | Nursing homes | EMS S&T | All care providers | All care providers / Hospitals – no data sharing (%) |
|---|---|---|---|---|---|---|
| 2012 | 256 | 368 | 35 | 15 | 578 | 226 |
| 2013 | 204 | 285 | 44 | 16 | 486 | 238 |
| 2014 | 261 | 395 | 25 | 19 | 706 | 270 |
| 2015 | 308 | 443 | 21 | 72 | 825 | 268 |
| 2016 | 344 | 511 | 30 | 47 | 845 | 246 |
| 2017 | 330 | 531 | 32 | 33 | 881 | 267 |

*Table 3. Number of frequent **users**, data on care provider categories, i.e., hospitals, nursing homes, EMS S&T, and all care providers, located in and outside Drenthe.*



For peer review only - http://bmjopen.bmj.com/site/about/guidelines.xhtml

| Year | Hospitals – no data sharing | Hospitals | Nursing homes | EMS S&T | All care providers | All care providers / Hospitals – no data sharing (%) |
|---|---|---|---|---|---|---|
| 2012 | 1984 | 2468 | 287 | 85 | 3826 | 193 |
| 2013 | 1829 | 2180 | 506 | 73 | 3658 | 200 |
| 2014 | 2120 | 2699 | 444 | 106 | 4685 | 221 |
| 2015 | 2116 | 2693 | 194 | 404 | 4902 | 232 |
| 2016 | 2515 | 3245 | 239 | 250 | 5228 | 208 |
| 2017 | 2220 | 3082 | 210 | 171 | 5133 | 231 |

*Table 4. Number of* ***calls*** *corresponding to frequent users, data on care provider categories, i.e., hospitals, nursing homes, EMS S&T, and all care providers, located in and outside Drenthe.*

For peer review only

## DISCUSSION

Tracing frequent users, i.e., patients displaying a high consumption (instead of appeal here and elsewhere) of health services, is considered highly relevant in regional policymaking. This is due to their high impact on care provider workload and use of scarce resources. The results of this study demonstrate that a networked approach for tracing frequent users relying on EMS data is capable of effectively and efficiently identifying frequent users. Case related results for the province of Drenthe indicate how more than a twofold frequent users may be traced by the proposed approach relative to existing approaches, relying on data collection by questioning individual care providers. Moreover, these results are obtained using a single source of data, whereas existing approaches would have required questioning a few hundred care providers.

Success of the proposed approach builds on its scope. As a straightforward effect of including more care providers, i.e., hospitals, nursing homes and EMS, located in and outside the region, more frequent users are traced and more accurately so. Most gains result from the possibility of combining data from different providers thus tracing those frequent users being network users, i.e., making use of multiple care providers, possibly representing inefficient use of scarce resources. Network users may easily be overlooked in existing approaches due to restrictions on their reach,



For peer review only - http://bmjopen.bmj.com/site/about/guidelines.xhtml

following from, for example, competition among care providers involved, incompatible information systems, and efforts to be put in data collection involving many providers. Case related results for the province of Drenthe indicate on average a 20% and 13% increase of the number of frequent users identified and their associated calls traced over the observation period, if hospital data were combined. Including hospitals outside Drenthe in this analysis improved the identification by another 28% and 52% on average. Extending scope to all categories of care providers, firstly focusing only on the Drenthe location, and secondly, setting no restrictions on care providers location, results in an increase of around 132% and 152% of frequent users identified. At the same time their associated calls increase by 123% and 114%, respectively. Gains found are relatively constant over the observation period. Relevance of being able to trace this group of network users follows from its expected growth among others resulting from on-going specialization in Dutch health care and outside. Moreover, their existence and upsurge may call for increased regional coordination among care providers to safeguard care continuity and avoid fragmented care and wrong referrals[11]. Clearly, being aware of frequent users is paramount to undertaking appropriate action.

The proposed networked approach may serve as a stepping stone in analysing consumption patterns of frequent users on a regional scale. Once frequent users have been identified by the approach, techniques such as process and data mining may allow for further group-wise analysis of patients routings along care providers, and their (joint) care needs following from EMS diagnostic data. These techniques have been successfully used to analyse healthcare processes, usually in an intra-hospital context [12-15]. Consumption patterns thus revealed may refine insights among policy makers on frequent users care needs, and their use of care services. In turn, revealing unfamiliar or much traversed patient routings may be helpful in, for example, optimizing these by



For peer review only - http://bmjopen.bmj.com/site/about/guidelines.xhtml

concerting activities among care providers or evoking patient treatment plans, thus improving and safeguarding quality of care.

The present study has limitations. Firstly, only EMS records for the province of Drenthe, The Netherlands are studied. Clearly, regional characteristics may have an effect on the results of the proposed approach. However, while this may be true, its success is not expected to be dependent on location or region, but relies on EMS' role as the regional ferryman and its records that oversee patient calls for service to a great many care providers. Secondly, EMS records only include frequent users who are not capable of self-transport. Thus, frequent users who do not, or rarely, make use of EMS will not be traced by the proposed approach. Thirdly, the success of any approach depends on the quality of the underlying data. We found how inclusion of ambulance transports to unknown, i.e., not recorded destinations in data analysis may result in higher numbers of frequent users and their associated calls being identified. Fourthly, as it is explorative, the paper signifies the potential of the proposed approach for tracing frequent users and enhancing regional policymaking. On-going and future research should be directed towards methodological issues concerning the use of the approach and its trade-off with alternative approaches.

For peer review only

## CONCLUSIONS

Many frequent users of care services are network users relying on multiple regional care providers, possibly representing inefficient use of scarce resources. Network users are effectively and efficiently traced by using EMS records offering high coverage of calls made to regional care providers.



For peer review only - http://bmjopen.bmj.com/site/about/guidelines.xhtml

## ACKNOWLEDGEMENTS

The authors would like to thank Mrs Harriëtte Holt from UMCG Ambulancezorg for providing input on the manuscript as domain expert, and Coert Schrijver for his technical support.

## Author Contributions

All authors contributed to the conception and design of the study, read and approved the submitted manuscript. LM contributed to data collection and data analysis, interpretation of the data and drafting the manuscript. DZ contributed to data analysis and interpretation of the data and drafting the manuscript. JH and EB contributed to the revision of the paper.

## REFERENCES

1. LaCalle E, Rabin E. Frequent users of emergency departments: the myths, the data, and the policy implications. *Ann Emerg Med* 2010;56(1):42-8. doi: 10.1016/j.annemergmed.2010.01.032 [published Online First: 2010/03/30]

2. Chan JS TA, Chow WL, Tiah L, Tiru M, Lee CE. Frequent attenders at the emergency department: an analysis of characteristics and utilisation trends. *Proceedings of Singapore Healthcare* 2018;27(1):12-19.

3. Cook LJ, Knight S, Junkins EP, Jr., et al. Repeat patients to the emergency department in a statewide database. *Acad Emerg Med* 2004;11(3):256-63. [published Online First: 2004/03/06]

4. Legramante J. Frequent Use of Emergency Departments by the Elderly Population When Continuing Care Is Not Well Established. *PLoS ONE* 2016;11(12)

5. Edwards MJ, Bassett, G., Sinden, L., Fothergill, R.T. . Frequent callers to the ambulance service: patient profiling and impact of case management on patient utilization of the ambulance service. *Emergency Medical Journal* 2014;32:392–96.



For peer review only - http://bmjopen.bmj.com/site/about/guidelines.xhtml

6. Scott J, Strickland AP, Warner K, et al. Describing and predicting frequent callers to an ambulance service: analysis of 1 year call data. *Emerg Med J* 2014;31(5):408-14. doi: 10.1136/emermed-2012-202146 [published Online First: 2013/02/16]

7. Eurostat. Eurostat - Population projections, 2019.

8. Rijksoverheid. Wet Maatschappelijke Ondersteuning, 2015.

9. Urbanoski K, Cheng J, Rehm J, et al. Frequent use of emergency departments for mental and substance use disorders. *Emerg Med J* 2018;35(4):220-25. doi: 10.1136/emermed-2015-205554

10. Seguin J, Osmanlliu E, Zhang X, et al. Frequent users of the pediatric emergency department. *CJEM* 2018;20(3):401-08. doi: 10.1017/cem.2017.15 [published Online First: 2017/04/07]

11. Dollard J, Harvey G, Dent E, et al. Older People Who Are Frequent Users of Acute Care: A Symptom of Fragmented Care? A Case Series Report on Patients' Pathways of Care. *J Frailty Aging* 2018;7(3):193-95. doi: 10.14283/10.14283/jfa.2018.12 [published Online First: 2018/08/11]

12. Mans RS, van der Aalst, W. M. P., Vanwersch, R. J. B. . Process Mining in Healthcare: Evaluating and Exploiting Operational Healthcare Processes: Springer 2015.

13. Rebuge A, Ferreira DR. Business process analysis in healthcare environments: A methodology based on process mining. *Information Systems* 2012;37(2):99-116. doi: 10.1016/j.is.2011.01.003

14. Rovani M, Maggi, F. M., de Leoni, M., & van der Aalst, W. M. P. . Declarative process mining in healthcare. *Expert Systems with Applications* 2015;42(23):9236–51.

15. Delias P, Doumpos M, Grigoroudis E, et al. Supporting healthcare management decisions via robust clustering of event logs. *Knowledge-Based Systems* 2015;84:203-13. doi: 10.1016/j.knosys.2015.04.012

For peer review only



For peer review only - http://bmjopen.bmj.com/site/about/guidelines.xhtml

BMJ Open

# Tracing frequent users of regional care services using emergency medical services data – a networked approach

| | |
|---:|---|
| Journal: | *BMJ Open* |
| Manuscript ID | bmjopen-2019-036139.R1 |
| Article Type: | Original research |
| Date Submitted by the Author: | 30-Mar-2020 |
| Complete List of Authors: | Maruster, Laura; University of Groningen Faculty of Economics and Business, IM&S<br>van der Zee, Durk-Jouke; Rijksuniversiteit Groningen Faculteit Economie en Bedrijfskunde, Operations<br>Hatenboer, Jaap; UMCG, Ambulancezorg<br>Buskens, Erik; University of Groningen, University Medical Center Groningen, Epidemiology |
| <b>Primary Subject Heading</b>: | Health services research |
| Secondary Subject Heading: | Emergency medicine, Research methods, Public health, Health policy |
| Keywords: | Health policy < HEALTH SERVICES ADMINISTRATION & MANAGEMENT, ACCIDENT & EMERGENCY MEDICINE, HEALTH ECONOMICS |
| | |

SCHOLARONE™ Manuscripts

For peer review only - http://bmjopen.bmj.com/site/about/guidelines.xhtml

*I, the Submitting Author has the right to grant and does grant on behalf of all authors of the Work (as defined in the below author licence), an exclusive licence and/or a non-exclusive licence for contributions from authors who are: i) UK Crown employees; ii) where BMJ has agreed a CC-BY licence shall apply, and/or iii) in accordance with the terms applicable for US Federal Government officers or employees acting as part of their official duties; on a worldwide, perpetual, irrevocable, royalty-free basis to BMJ Publishing Group Ltd ("BMJ") its licensees and where the relevant Journal is co-owned by BMJ to the co-owners of the Journal, to publish the Work in this journal and any other BMJ products and to exploit all rights, as set out in our licence.*

*The Submitting Author accepts and understands that any supply made under these terms is made by BMJ to the Submitting Author unless you are acting as an employee on behalf of your employer or a postgraduate student of an affiliated institution which is paying any applicable article publishing charge ("APC") for Open Access articles. Where the Submitting Author wishes to make the Work available on an Open Access basis (and intends to pay the relevant APC), the terms of reuse of such Open Access shall be governed by a Creative Commons licence – details of these licences and which Creative Commons licence will apply to this Work are set out in our licence referred to above.*

*Other than as permitted in any relevant BMJ Author's Self Archiving Policies, I confirm this Work has not been accepted for publication elsewhere, is not being considered for publication elsewhere and does not duplicate material already published. I confirm all authors consent to publication of this Work and authorise the granting of this licence.*

For peer review only

# Tracing frequent users of regional care services using emergency medical services data – a networked approach

Laura Maruster[1] PhD, Durk-Jouke van der Zee[2] PhD, Jaap Hatenboer[3], Erik Buskens[2,4] MD, PhD.

*[1] Department of Innovation Management & Strategy, Faculty of Economics & Business, University of Groningen, The Netherlands*

*[2] Department of Operations, Faculty of Economics & Business, University of Groningen, The Netherlands*

*[3] UMCG Ambulancezorg, Tynaarlo, The Netherlands*

*[4] Health Technology Assessment, Department of Epidemiology, University Medical Center Groningen, University of Groningen, Groningen, The Netherlands*

Key words: regional care services, hospital care, nursing care, emergency medical services, frequent users

* Corresponding author: Laura Maruster, Nettelbosje 2, P.O. Box 800, 9700 AV Groningen, The Netherlands. Phone + 31 50 363 7316, email: l.maruster@rug.nl

Word count: 2840



For peer review only - http://bmjopen.bmj.com/site/about/guidelines.xhtml

## ABSTRACT

**Objectives**

This study shows how a networked approach relying on 'real-world' Emergency Medical Services (EMS) records might contribute to tracing frequent users of care services on a regional scale. Their tracing is considered of importance for policy makers and clinicians, since they represent a considerable workload and use of scarce resources. While existing approaches for data collection on frequent users tend to limit scope to individual or associated care providers, the proposed approach exploits the role of EMS as the network's "ferryman" overseeing and recording patient calls made to an entire network of care providers.

**Design**

A retrospective study was performed analysing 2012-2017 EMS calls in the province of Drenthe, -the Netherlands. Using EMS data benefits of the networked approach vs. existing approaches are assessed by quantifying the number of frequent users and their associated calls for various categories of care providers. Main categories considered are hospitals, nursing homes and EMS.

**Setting**

EMS in the province of Drenthe, the Netherlands, serving a population of 491,867.

**Participants**

Analyses are based on secondary patient data from EMS records, entailing 212,967 transports and 126,758 patients, over 6 years (2012-2017).

**Results**

Use of the networked approach for analysing calls made to hospitals in Drenthe resulted in a 20% average increase of frequent users traced. Extending the analysis by including hospitals outside Drenthe increased ascertainment by 28%. Extending to all categories of care providers, inside Drenthe, and subsequently, irrespective of their location, resulted in an average increase of 132% and 152% of frequent users identified, respectively.


For peer review only



For peer review only - http://bmjopen.bmj.com/site/about/guidelines.xhtml

**Conclusions**

Many frequent users of care services are network users relying on multiple regional care providers, possibly representing inefficient use of scarce resources. Network users are effectively and efficiently traced by using EMS records offering high coverage of calls made to regional care providers.

For peer review only



For peer review only - http://bmjopen.bmj.com/site/about/guidelines.xhtml

## ARTICLE SUMMARY

### Strengths and limitations of this study

- Single source comprehensive data such as Emergency Medical Services (EMS)' serving an entire region allows identification and tracing of frequent users of health services.
- EMS data include an extensive collection of patient transport data relating subsequent services provided by hospitals and nursing homes.
- A networked approach for tracing frequent users relying on EMS records, allowed us to effectively and efficiently identify frequent users.
- We piloted EMS records for only one province in the Netherlands, while we expect that this generic approach could be easily transposed to any region.
- As EMS records only include frequent users who are not capable of self-transport, those frequent users who do not, or rarely make use of EMS will not be traced by the proposed approach.

For peer review only

## INTRODUCTION

Frequent users, i.e., patients that make repetitive calls for health care services, may be responsible for a relatively large share of regional care consumption. They represent a minority of Emergency Department (ED) patients (4.5-8%), yet, they may account for up to 21-28% of all ED visits[1-3]. Different solutions have been devised for frequent users once identified. Subsequently, the appropriate answers to their needs, and consequently reducing the visits to ED and ambulance transports may be achieved. These solutions range from case management [4-6], to individual care plans[7-9], and facilitated contacts with healthcare providers[10]. However, to be able to offer and consider such a form of advance care planning for apparently frail patients they first need to be identified. The latter in reality may escape attention or appear difficult with data scattered over various institutions. Clearly, due to their high impact on care providers' workload and associated costs they are a focal group for regional policy makers and clinicians aiming to make best use of scarce resources. In the Netherlands and possibly other settings the Emergency Medical Services (EMS) are increasingly overburdened, and at times encounter backlogs at the EDs of hospitals[11 12]. Indeed, the role of EMS in triage and adequate and timely referral is increasingly recognised in acute care networks. Accordingly, identifying opportunities to relieve an overburdened acute care system from frequent and inappropriate may be considered an impending responsibility of EMS.

Notably, many frequent users appear to be network users, relying on multiple care providers [13]. Their choice of care providers is influenced by, for example, their preferences and care providers' specialization. In particular, tracing patients' network use tends to be cumbersome. Hurdles not easily taken in data collection are, for example, rules on patient privacy, competition among care providers, incompatibility of information systems, and efforts to be put in. Moreover, these hurdles likely imply high data collection costs. Not surprisingly, many research designs limit their scope



For peer review only - http://bmjopen.bmj.com/site/about/guidelines.xhtml

to single or associated care providers, with a main focus on hospitals (see for example [2 13-29] ). Hence, many factual frequent users may remain unnoticed.

Basically, current approaches towards data collection on frequent users stress probing of individual care providers [23 30]. Alternatively, acknowledging frequent users being network users, this article suggests a networked approach for their tracing, relying on EMS data. Acting as the “ferryman” in the regional network, EMS oversee and record patient calls made to regional care providers, including hospitals and nursing homes. Importantly, the EMS patient population is likely to include many frequent users [31 32]. In addition, their need for mobile nursing services and transport indicates that their requirements of care resources may be high.

The aim of the present study is to show how the use of the proposed networked approach might efficiently contribute to tracing frequent users on a regional scale.

## METHODS

### Care network in the province of Drenthe

The province of Drenthe, the Netherlands, has a population of 491,867 inhabitants, with a population density of 183 inhabitants per square kilometer[33]. Hospital care for its population is provided by four hospitals within the province, and by several hospitals located in neighbouring provinces. Three of the hospitals in Drenthe offer basic treatment. In one hospital the necessary skills and resources for treating multi-level traumas are present. Referral to around 80 hospitals in other provinces is motivated by reasons such as their proximity to the patient scene, patient preferences, level of care or specialization in specific treatments. Nursing care is provided by a few dozen of large homes, and around three hundred smaller (specialized) homes, mainly located within the province. EMS is provided by a single operator, relying on a network of 14 bases in 13 cities/villages in Drenthe. Its services include both urgent and planned patient transports to



For peer review only - http://bmjopen.bmj.com/site/about/guidelines.xhtml

hospitals and planned transports to nursing homes. Planned rides are legitimated by patient care needs that prohibit self-transport.

**Data**

Patient data are collected from EMS records of ambulance rides performed between January 1st 2012 – December 31st 2017. Collected data include the rides' dates and times, and destinations, i.e., care providers. EMS is marked as a formal care provider in case treatment provided by the ambulance nurse on scene suffices to address patient care needs, i.e., EMS See & Treat (EMS S&T), implying no involvement of other care providers. Motivated by EMS scope of services, three categories of care providers are distinguished, i.e., hospitals, nursing homes and EMS S&T.

**Privacy and approval**. Since the data are routinely collected for administrative purposes, and completely anonymized, i.e., there is no direct contact with identifiable persons, this study does not fall within the scope of the Medical Research Involving Human Subjects Act (WMO) [34]. We obtained a full waiver for using anonymized data from the EMS services from our institutional ethical review board.

**Patient and public involvement.** No patient and public involved.

**Data analysis**

For data analysis, EMS records referring to single rides are anonymized, cleaned by removing empty records, i.e., records not relating to patients, and inspected for correctness of data provided. Patients qualify as a frequent user if they meet a threshold of four calls in a calendar year. Although definitions differ, usually a threshold of four to five calls or more per year is used to classify a



For peer review only - http://bmjopen.bmj.com/site/about/guidelines.xhtml

patient as a 'frequent user' [35 36]. Frequent users are quantified by presenting their numbers and number of calls, including yearly trend figures.

The potential of the proposed network-based approach for data collection on frequent users is evaluated by assessing its benefits compared to existing approaches. Whereas the proposed approach relies on EMS data, existing approaches build on data obtained from individual care providers. In principle, both approaches may render similar outcomes. However, existing approaches face hurdles not easily overcome, due to the fact that multiple organizations, i.e., care providers, are involved in data collection. Known hurdles are rules on patient privacy, competition among care providers, incompatibility of information systems, efforts to be put in, and costs of overcoming hurdles. They likely restrict the scope of data collection, i.e., the number of care providers being considered. Restriction of scope may affect identifying patients making calls to various care providers as frequent users after combining and quantifying their calls. The proposed approach relies on a single source of data, and does overcome these scoping decisions.

Effects of the choice of scope on the number of frequent users identified and their associated calls are studied by considering alternative subsets of EMS records. Choice of subsets is related to provider categories, i.e., hospitals, nursing homes, and/or EMS S&T, and their location, i.e., inside or outside Drenthe. By either allowing patient records to be combined for chosen subsets of care providers, or not, beneficial effects of the possibility to identify network users are assessed. This effect is studied for hospitals, serving most of the patient calls.

## RESULTS

EMS records for 2012-2017, refer to 212,967 calls for services, involving 126,758 patients. Data cleaning resulted in 2,494 calls being removed. In addition, 13,156 calls (6%) were discarded due



For peer review only - http://bmjopen.bmj.com/site/about/guidelines.xhtml

to unknown, not recorded destinations (i.e. care providers). The remaining 199,811 calls are included in the study. Out of these 199,811 calls 147,027 (74%), 10,976 (5%), and 41,808 (21%) refer to services provided by hospitals, nursing homes, and EMS S&T, respectively. Results of the evaluation of the networked approach for data collection on frequent users are shown in Tables 1-4. Tables 1 and 2 quantify the number of *frequent users* and their associated *calls* for alternative choices of categories of care providers located in Drenthe on a yearly basis. Categories of care providers considered are hospitals, nursing homes, EMS S&T, and all care providers, i.e., taking all aforementioned categories together. Except for hospitals, all results assume data sharing among care providers within categories set, allowing frequent users being network users to be traced. In addition, hospitals results are shown for settings where such data sharing among single hospitals is not possible. Hence, network users may be neglected. For respective settings, the number of unique frequent users is shown, i.e., numbers are corrected for the fact that a single patient may be classified as a frequent user for multiple hospitals. The final column indicates the effect of combining data for all care providers vs. a setting where frequent users of hospital services are identified by studying single hospitals in isolation. It shows how the number of frequent users traced and their associated calls increase by more than a twofold by combining data for all care providers. Similar to Tables 1 and 2, Tables 3 and 4 quantify the annual number of *frequent users* and their associated *calls* for alternative choices of categories of care providers, without setting requirements to their location. Final columns in Tables 3 and 4 show which numbers of frequent users and their associated calls are found when using the full EMS data set, including care providers located outside Drenthe. These amount to around 2.5 times the numbers found when studying hospitals in isolation.



For peer review only - http://bmjopen.bmj.com/site/about/guidelines.xhtml

| Year | Hospitals - no data sharing | Hospitals | Nursing homes | EMS S&T | All care providers | All care providers / Hospitals – no data shared (%) |
|---|---|---|---|---|---|---|
| 2012 | 189 | 222 | 34 | 15 | 398 | 211 |
| 2013 | 153 | 181 | 42 | 16 | 340 | 222 |
| 2014 | 204 | 245 | 22 | 19 | 495 | 243 |
| 2015 | 253 | 309 | 18 | 68 | 635 | 251 |
| 2016 | 279 | 321 | 28 | 46 | 611 | 219 |
| 2017 | 263 | 332 | 30 | 33 | 649 | 247 |

*Table 1. Number of frequent **users**, using data on care provider categories, i.e., hospitals, nursing homes, EMS S&T, and all care providers, located in Drenthe.*

| Year | Hospitals - no data sharing | Hospitals | Nursing homes | EMS S&T | All care providers | All care providers / Hospitals – no data shared (%) |
|---|---|---|---|---|---|---|
| 2012 | 1161 | 1296 | 283 | 84 | 2423 | 209 |
| 2013 | 1158 | 1279 | 497 | 73 | 2503 | 216 |
| 2014 | 1386 | 1557 | 431 | 106 | 3204 | 231 |
| 2015 | 1477 | 1711 | 174 | 388 | 3597 | 244 |
| 2016 | 1772 | 1955 | 229 | 245 | 3631 | 205 |
| 2017 | 1536 | 1821 | 193 | 170 | 3581 | 233 |

*Table 2. Number of **calls** corresponding to frequent users, using data on care provider categories, i.e., hospitals, nursing homes, EMS S&T, and all care providers, located in Drenthe.*

| Year | Hospitals – no data sharing | Hospitals | Nursing homes | EMS S&T | All care providers | All care providers / Hospitals – no data sharing (%) |
|---|---|---|---|---|---|---|
| 2012 | 256 | 368 | 35 | 15 | 578 | 226 |
| 2013 | 204 | 285 | 44 | 16 | 486 | 238 |
| 2014 | 261 | 395 | 25 | 19 | 706 | 270 |
| 2015 | 308 | 443 | 21 | 72 | 825 | 268 |
| 2016 | 344 | 511 | 30 | 47 | 845 | 246 |
| 2017 | 330 | 531 | 32 | 33 | 881 | 267 |

*Table 3. Number of frequent **users**, data on care provider categories, i.e., hospitals, nursing homes, EMS S&T, and all care providers, located in and outside Drenthe.*

| Year | Hospitals – no data sharing | Hospitals | Nursing homes | EMS S&T | All care providers | All care providers / Hospitals – no data sharing (%) |
|---|---|---|---|---|---|---|
| 2012 | 1984 | 2468 | 287 | 85 | 3826 | 193 |
| 2013 | 1829 | 2180 | 506 | 73 | 3658 | 200 |
| 2014 | 2120 | 2699 | 444 | 106 | 4685 | 221 |
| 2015 | 2116 | 2693 | 194 | 404 | 4902 | 232 |
| 2016 | 2515 | 3245 | 239 | 250 | 5228 | 208 |



For peer review only - http://bmjopen.bmj.com/site/about/guidelines.xhtml

For peer review only

| | | | | | | |
|---|---|---|---|---|---|---|
| 2017 | 2220 | 3082 | 210 | 171 | 5133 | 231 |

*Table 4. Number of **calls** corresponding to frequent users, data on care provider categories, i.e., hospitals, nursing homes, EMS S&T, and all care providers, located in and outside Drenthe.*

## DISCUSSION

Tracing frequent users, i.e., patients displaying a high consumption (instead of appeal here and elsewhere) of health services, is considered highly relevant in regional policymaking. This is due to their high impact on care provider workload and use of scarce resources. The results of this study demonstrate that a networked approach for tracing frequent users relying on EMS data is capable of effectively and efficiently identifying frequent users. Case related results for the province of Drenthe indicate how more than a twofold frequent users may be traced by the proposed approach relative to existing approaches, relying on data collection by questioning individual care providers. Moreover, these results are obtained using a single source of data, whereas existing approaches would have required questioning a few hundred care providers.

Success of the proposed approach builds on its scope. As a straightforward effect of including more care providers, i.e., hospitals, nursing homes and EMS, located in and outside the region, more frequent users are traced and more accurately so. Most gains result from the possibility of combining data from different providers thus tracing those frequent users being network users, i.e., making use of multiple care providers, possibly representing inefficient use of scarce resources. Network users may easily be overlooked in existing approaches due to restrictions on their reach, following from, for example, competition among care providers involved, incompatible information systems, and efforts to be put in data collection involving many providers. Case related results for the province of Drenthe indicate on average a 20% and 13% increase of the number of frequent users identified and their associated calls traced over the observation period, if hospital

For peer review only

data were combined. Including hospitals outside Drenthe in this analysis improved the identification by another 28% and 52% on average. Extending scope to all categories of care providers, firstly focusing only on the Drenthe location, and secondly, setting no restrictions on care providers location, results in an increase of around 132% and 152% of frequent users identified. At the same time their associated calls increase by 123% and 114%, respectively. Gains found are relatively constant over the observation period. Relevance of being able to trace this group of network users follows from its expected growth among others resulting from on-going specialization in Dutch health care and outside. Moreover, their existence and upsurge may call for increased regional coordination among care providers to safeguard care continuity and avoid fragmented care and wrong referrals[37]. Clearly, being aware of frequent users is paramount to undertaking appropriate action. The opportunity we identified and seized might seem trivial in settings where individuals are easily traced, i.e., single payer or service provider systems. In these systems the necessity to take appropriate action is no less urgent, yet the effort to obtain a listing and pattern of use might be simpler. Nevertheless, we provide a worked out exemplary approach that may be applied in many settings like the Netherlands.

The proposed networked approach may serve as a stepping stone in analysing consumption patterns of frequent users on a regional scale. Once frequent users have been identified by the approach, techniques such as process and data mining may allow for further group-wise analysis of patients routings along care providers, and their (joint) care needs following from EMS diagnostic data. These techniques have been successfully used to analyse healthcare processes, usually in an intra-hospital context [38-41]. Whereas process mining may be helpful in capturing patients' routing along care providers, data mining may assist in analysing patients' care needs further using text analysis of diagnostic data, thereby unravelling their reasons for calls. Consumption patterns thus revealed may refine insights among policy makers on frequent users



For peer review only - http://bmjopen.bmj.com/site/about/guidelines.xhtml

care needs, and their use of care services. In turn, revealing unfamiliar or much traversed patient routings may be helpful in, for example, optimizing these by concerting activities among care providers or evoking patient treatment plans, thus improving and safeguarding quality of care.

The present study has limitations. Firstly, only EMS records for the province of Drenthe, the Netherlands are studied. Clearly, regional characteristics may have an effect on the results of the proposed approach. However, while this may be true, its success is not expected to be dependent on location or region, but relies on EMS' role as the regional ferryman and its records that oversee patient calls for service to a great many care providers. Secondly, EMS records only include frequent users who are not capable of self-transport. Thus, frequent users who do not, or rarely, make use of EMS will not be traced by the proposed approach. Thirdly, the success of any approach depends on the quality of the underlying data. We found how inclusion of ambulance transports to unknown, i.e., not recorded destinations in data analysis may result in higher numbers of frequent users and their associated calls being identified. Fourthly, as it is explorative, the paper signifies the potential of the proposed approach for tracing frequent users and enhancing regional policymaking. On-going and future research should be directed towards methodological issues concerning the use of the approach and its trade-off with alternative approaches.

## CONCLUSIONS

Many frequent users of care services are network users relying on multiple regional care providers, possibly representing inefficient use of scarce resources. Network users are effectively and efficiently traced by using EMS records offering high coverage of calls made to regional care providers.



For peer review only - http://bmjopen.bmj.com/site/about/guidelines.xhtml

For peer review only

## ACKNOWLEDGEMENTS

The authors would like to thank Mrs Harriëtte Holt from UMCG Ambulancezorg for providing input on the manuscript as domain expert, and Coert Schrijver for his technical support.

### Author Contributions

All authors contributed to the conception and design of the study, read and approved the submitted manuscript. LM contributed to data collection and data analysis, interpretation of the data and drafting the manuscript. DZ contributed to data analysis, interpretation of the data and drafting the manuscript. JH and EB contributed to data analysis and interpretation of the data and to the revision of the paper.

### Competing interests

There are no competing interests for any author.

### Funding

There is no funding to report for this submission.

### Data sharing

No additional data are available.

## REFERENCES



For peer review only - http://bmjopen.bmj.com/site/about/guidelines.xhtml

1. Hudon C, Courteau J, Krieg C, et al. Factors associated with chronic frequent emergency department utilization in a population with diabetes living in metropolitan areas: a population-based retrospective cohort study. *BMC Health Serv Res* 2017;17(1):525. doi: 10.1186/s12913-017-2453-3 [published Online First: 2017/08/06]

2. Doupe MB, Palatnick W, Day S, et al. Frequent Users of Emergency Departments: Developing Standard Definitions and Defining Prominent Risk Factors. *Annals of Emergency Medicine* 2012;60(1):24-32. doi: 10.1016/j.annemergmed.2011.11.036

3. LaCalle E, Rabin E. Frequent Users of Emergency Departments: The Myths, the Data, and the Policy Implications. *Annals of Emergency Medicine* 2010;56(1):42-48. doi: 10.1016/j.annemergmed.2010.01.032

4. Hudon C, Chouinard MC, Pluye P, et al. Characteristics of Case Management in Primary Care Associated With Positive Outcomes for Frequent Users of Health Care: A Systematic Review. *Annals of Family Medicine* 2019;17(5):448-58. doi: 10.1370/afm.2419

5. Moschetti K, Iglesias K, Baggio S, et al. Health care costs of case management for frequent users of the emergency department: Hospital and insurance perspectives. *Plos One* 2018;13(9) doi: 10.1371/journal.pone.0199691

6. Grover CA, Sughair J, Stoopes S, et al. Case Management Reduces Length of Stay, Charges, and Testing in Emergency Department Frequent Users. *Western Journal of Emergency Medicine* 2018;19(2):238-44. doi: 10.5811/westjem.2017.9.34710

7. Moe J, Kirkland SW, Rawe E, et al. Effectiveness of Interventions to Decrease Emergency Department Visits by Adult Frequent Users: A Systematic Review. *Academic Emergency Medicine* 2017;24(1):40-52. doi: 10.1111/acem.13060

8. Pope D, Fernandes CMB, Bouthillette F, et al. Frequent users of the emergency department: a program to improve care and reduce visits. *Canadian Medical Association Journal* 2000;162(7):1017-20.

9. Spillane LL, Lumb EW, Cobaugh DJ, et al. Frequent users of the emergency department: Can we intervene? *Academic Emergency Medicine* 1997;4(6):574-80. doi: 10.1111/j.1553-2712.1997.tb03581.x



For peer review only - http://bmjopen.bmj.com/site/about/guidelines.xhtml

10. Agarwal G, Pirrie M, McLeod B, et al. Rationale and methods of an Evaluation of the Effectiveness of the Community Paramedicine at Home (CP@home) program for frequent users of emergency medical services in multiple Ontario regions: a study protocol for a randomized controlled trial. *Trials* 2019;20 doi: 10.1186/s13063-018-3107-4

11. Backer HD, D'Arcy NT, Davis AJ, et al. Statewide Method of Measuring Ambulance Patient Offload Times. *Prehosp Emerg Care* 2019;23(3):319-26. doi: 10.1080/10903127.2018.1525456 [published Online First: 2018/09/28]

12. Cooney DR, Millin MG, Carter A, et al. Ambulance diversion and emergency department offload delay: resource document for the National Association of EMS Physicians position statement. *Prehosp Emerg Care* 2011;15(4):555-61. doi: 10.3109/10903127.2011.608871 [published Online First: 2011/08/30]

13. Castillo EM, Brennan JJ, Killeen JP, et al. IDENTIFYING FREQUENT USERS OF EMERGENCY DEPARTMENT RESOURCES. *Journal of Emergency Medicine* 2014;47(3):343-47. doi: 10.1016/j.jemermed.2014.03.014

14. Chan JS TA, Chow WL, Tiah L, Tiru M, Lee CE. Frequent attenders at the emergency department: an analysis of characteristics and utilisation trends. *Proceedings of Singapore Healthcare* 2018;27(1):12-19.

15. Levola JM, Sailas ES, Saamanen TS, et al. A register-based observational cohort study on persistent frequent users of emergency services in a Finnish emergency clinic. *Bmc Health Services Research* 2019;19(1) doi: 10.1186/s12913-019-4723-8

16. Birmingham LE, Cheruvu VK, Frey JA, et al. Distinct subgroups of emergency department frequent users: A latent class analysis. *American Journal of Emergency Medicine* 2020;38(1):83-88. doi: 10.1016/j.ajem.2019.04.029

17. Kuek BJW, Li HH, Yap S, et al. Characteristics of Frequent Users of Emergency Medical Services in Singapore. *Prehospital Emergency Care* 2019;23(2):215-24. doi: 10.1080/10903127.2018.1484969

For peer review only



For peer review only - http://bmjopen.bmj.com/site/about/guidelines.xhtml

18. Bertoli-Avella AM, Haagsma JA, Van Tiel S, et al. Frequent users of the emergency department services in the largest academic hospital in the Netherlands: a 5-year report. *European Journal of Emergency Medicine* 2017;24(2):130-35. doi: 10.1097/mej.0000000000000314

19. Seguin J, Osmanlliu E, Zhang X, et al. Frequent users of the pediatric emergency department. *Canadian Journal of Emergency Medicine* 2018;20(3):401-08. doi: 10.1017/cem.2017.15

20. Dollard J, Harvey G, Dent E, et al. OLDER PEOPLE WHO ARE FREQUENT USERS OF ACUTE CARE: A SYMPTOM OF FRAGMENTED CARE? A CASE SERIES REPORT ON PATIENTS' PATHWAYS OF CARE. *Journal of Frailty & Aging* 2018;7(3):193-95. doi: 10.14283/jfa.2018.12

21. Meng XF, Muggli T, Baetz M, et al. Disordered lives: Life circumstances and clinical characteristics of very frequent users of emergency departments for primary mental health complaints. *Psychiatry Research* 2017;252:9-15. doi: 10.1016/j.psychres.2017.02.044

22. Norman C, Mello M, Choi B. Identifying Frequent Users of an Urban Emergency Medical Service Using Descriptive Statistics and Regression Analyses. *Western Journal of Emergency Medicine* 2016;17(1):39-45. doi: 10.5811/westjem.2015.10.28508

23. Legramante JM, Morciano L, Lucaroni F, et al. Frequent Use of Emergency Departments by the Elderly Population When Continuing Care Is Not Well Established. *Plos One* 2016;11(12) doi: 10.1371/journal.pone.0165939

24. Vu F, Daeppen JB, Hugli O, et al. Screening of mental health and substance users in frequent users of a general Swiss emergency department. *Bmc Emergency Medicine* 2015;15 doi: 10.1186/s12873-015-0053-2

25. Acosta AM, Lima M. Frequent users of emergency services: associated factors and reasons for seeking care. *Revista Latino-Americana De Enfermagem* 2015;23(2):337-44. doi: 10.1590/0104-1169.0072.2560

26. Bodenmann P, Daeppen JB, Vaucher P, et al. SOCIAL AND MEDICAL VULNERABILITY FACTORS OF FREQUENT USERS AT THE EMERGENCY DEPARTMENT: A CASE-CONTROL STUDY. *Journal of General Internal Medicine* 2010;25:391-92.



For peer review only - http://bmjopen.bmj.com/site/about/guidelines.xhtml

27. Geurts J, Palatnick W, Strome T, et al. Frequent users of an inner-city emergency department. *Canadian Journal of Emergency Medicine* 2012;14(5):306-13. doi: 10.2310/8000.2012.120670

28. Bieler G, Paroz S, Faouzi M, et al. Social and Medical Vulnerability Factors of Emergency Department Frequent Users in a Universal Health Insurance System. *Academic Emergency Medicine* 2012;19(1):63-68. doi: 10.1111/j.1553-2712.2011.01246.x

29. Locker TE, Baston S, Mason SM, et al. Defining frequent use of an urban emergency department. *Emergency Medicine Journal* 2007;24(6):398-401. doi: 10.1136/emj.2006.043844

30. Cook LJ, Knight S, Junkins EP, Jr., et al. Repeat patients to the emergency department in a statewide database. *Acad Emerg Med* 2004;11(3):256-63. [published Online First: 2004/03/06]

31. Edwards MJ, Bassett, G., Sinden, L., Fothergill, R.T. . Frequent callers to the ambulance service: patient profiling and impact of case management on patient utilization of the ambulance service. *Emergency Medical Journal* 2014;32:392–96.

32. Scott J, Strickland AP, Warner K, et al. Describing and predicting frequent callers to an ambulance service: analysis of 1 year call data. *Emerg Med J* 2014;31(5):408-14. doi: 10.1136/emermed-2012-202146 [published Online First: 2013/02/16]

33. Eurostat. Eurostat - Population projections, 2019.

34. Rijksoverheid. Wet Maatschappelijke Ondersteuning, 2015.

35. Urbanoski K, Cheng J, Rehm J, et al. Frequent use of emergency departments for mental and substance use disorders. *Emergency Medicine Journal* 2018;35(4):220-25. doi: 10.1136/emermed-2015-205554

36. Seguin J, Osmanlliu E, Zhang X, et al. Frequent users of the pediatric emergency department. *CJEM* 2018;20(3):401-08. doi: 10.1017/cem.2017.15 [published Online First: 2017/04/07]

37. Dollard J, Harvey G, Dent E, et al. Older People Who Are Frequent Users of Acute Care: A Symptom of Fragmented Care? A Case Series Report on Patients' Pathways of Care. *J Frailty Aging* 2018;7(3):193-95. doi: 10.14283/10.14283/jfa.2018.12 [published Online First: 2018/08/11]

38. Mans RS, van der Aalst, W. M. P., Vanwersch, R. J. B. . Process Mining in Healthcare: Evaluating and Exploiting Operational Healthcare Processes: Springer 2015.

For peer review only



For peer review only - http://bmjopen.bmj.com/site/about/guidelines.xhtml

39. Rebuge A, Ferreira DR. Business process analysis in healthcare environments: A methodology based on process mining. *Information Systems* 2012;37(2):99-116. doi: 10.1016/j.is.2011.01.003

40. Rovani M, Maggi, F. M., de Leoni, M., & van der Aalst, W. M. P. . Declarative process mining in healthcare. *Expert Systems with Applications* 2015;42(23):9236–51.

41. Delias P, Doumpos M, Grigoroudis E, et al. Supporting healthcare management decisions via robust clustering of event logs. *Knowledge-Based Systems* 2015;84:203-13. doi: 10.1016/j.knosys.2015.04.012

For peer review only



For peer review only - http://bmjopen.bmj.com/site/about/guidelines.xhtml
